# Inactivated Rabies Virus-Vectored Immunocontraceptive Vaccine in a Thermo-Responsive Hydrogel Induces High and Persistent Antibodies against Rabies, but Insufficient Antibodies against Gonadotropin-Releasing Hormone for Contraception

**DOI:** 10.3390/vaccines7030073

**Published:** 2019-07-25

**Authors:** Xianfu Wu, Yong Yang, Chantal Kling, Laurie Seigler, Nadia F. Gallardo-Romero, Brock E. Martin, Todd G. Smith, Victoria A. Olson

**Affiliations:** 1 Centers for Disease Control and Prevention, Poxvirus and Rabies Branch/DHCPP/NCEZID, Atlanta, GA 30329, USA; 2 ARK Temporary Staffing, Lawrenceville, GA 30046, USA; 3 Oak Ridge Institute for Science and Education, Oak Ridge, TN 37830, USA

**Keywords:** gonadotropin-releasing hormone (GnRH), immunocontraceptive vaccine, rabies virus, ERA-2GnRH, chitosan, thermo-responsive hydrogel, nonsurgical sterilization

## Abstract

Rabies is preventable through vaccination, but the need to mount annual canine vaccination campaigns presents major challenges in rabies control and prevention. The development of a rabies vaccine that ensures lifelong immunity and animal population management in one dose could be extremely advantageous. A nonsurgical alternative to spay/neuter is a high priority for animal welfare, but irreversible infertility in one dose has not been achieved. Towards this goal, we developed a rabies virus-vectored immunocontraceptive vaccine ERA-2GnRH, which protected against rabies virus challenge and induced >80% infertility in mice after three doses in a live, liquid-vaccine formulation (Wu et al., 2014). To improve safety and use, we formulated an inactivated vaccine in a thermo-responsive chitosan hydrogel for one-dose delivery and studied the immune responses in mice. The hydrogel did not cause any injection site reactions, and the killed ERA-2GnRH vaccine induced high and persistent rabies virus neutralizing antibodies (rVNA) in mice. The rVNA in the hydrogel group reached an average of 327.40 IU/mL, more than 200 times higher than the liquid vaccine alone. The Gonadotropin-releasing hormone (GnRH) antibodies were also present and lasted longer in the hydrogel group, but did not prevent fertility in mice, reflecting a possible threshold level of GnRH antibodies for contraception. In conclusion, the hydrogel facilitated a high and long-lasting immunity, and ERA-2GnRH is a promising dual vaccine candidate. Future studies will focus on rabies protection in target species and improving the anti-GnRH response.

## 1. Introduction

To control rabies in dogs, vaccination is the key strategy. Dog rabies vaccine is one of the “core vaccines” in veterinary practice, and the vaccination schedule should be strictly followed [1]. A dog receives its first rabies vaccination at 3 months or older, is boosted after a year, and is then revaccinated every year or every 3 years thereafter, depending on how the vaccine is registered and licensed [2]. For a dog living to 15 years, there will be 6 to 16 veterinary clinical visits and vaccinations to meet the requirement. If there is a skip or noncompliance during the schedule, vaccination will be reevaluated or reinitiated. This practice is costly and requires a high level of responsible dog ownership. Rabies has been well controlled in many countries through this practice. Japan’s experience is a good example of achieving rabies-free status in a country. From 1914 to 1921 when no rabies vaccine was available for dogs, Osaka Prefecture controlled rabies transmission through the capture of stray dogs and leashing of pet dogs [3]. Therefore, human behavior and dog behavior are both important in rabies control. In developing countries where rabies is enzootic, many dogs are owned but free roaming [4]. Mass dog vaccination campaigns are mostly ad hoc events [5,6,7,8] and could not achieve the “standard dog vaccination schedule” of vaccinating the same dog every year or every 3 years. In order to provide an option for rabies control under those conditions, we have been working on the development of a dual vaccine for long-lasting rabies protection and animal population management [9,10,11]. The ultimate goal of our research is to protect against rabies in dogs with only one vaccination and simultaneously mitigate reproductive behaviors in free-roaming dogs. The goal is ambitious, and has not been intensively explored due to technical challenges.

Here, we presented the continuation of our studies relating to the development of an effective vaccine for both protection from rabies and prevention of reproduction [11]. For the first trial, the vaccine (ERA-2GnRH) was formulated in chitosan hydrogel and delivered to mice in one dose. Thermo-responsive hydrogels stay in liquid form at room temperature (R/T, 22 °C ± 3 °C) and turn into a semi-solid gel when delivered to animals (37 °C). The vaccine formulation was biodegradable, caused no adverse effects at the injection site, and induced high and long-lasting immune responses against both rabies and the gonadotropin-releasing hormone (GnRH). Although the vaccine did not prevent fertility after one-dose vaccination in mice, we gained important insight into the threshold level of GnRH antibodies required for contraception. The study demonstrated that the vaccine release profile was controlled by different formulations. Further studies will help us understand how infertility is achieved by a single ERA-2GnRH vaccination. Another interesting and important finding in the study was that the tedious dog vaccination regimen could be simplified. Future studies will explore whether rabies vaccine in hydrogels induces life-long protection in animals by one-dose vaccination.

## 2. Materials and Methods

### 2.1. Cells, ERA-2GnRH Virus Growth and Concentration

The recombinant rabies virus (RABV) ERA-2GnRH has been described previously [10,11]. The BSR cells (a clone of baby hamster kidney cell line) were grown in the Dulbecco’s minimal essential medium (DMEM) supplemented with 10% fetal bovine serum, 10,000 unit/mL of penicillin, 10,000 µg/mL of streptomycin, and 25 µg/mL of Fungizone (CDC, Division of Scientific Resources). The cells at 1.0 × 10^6^ /mL were seeded in a T75 culture flask and infected simultaneously with ERA-2GnRH with a multiplicity of infection of 1.0. Four to 5 days after infection, the cell supernatants were sampled for virus titration as described previously [12]. The virus was harvested when the titer reached 1.0 × 10^7^ focus forming units (ffu)/mL or higher. After centrifugation at 1500× *g* for 15 min to remove cell debris, the virus supernatants were further filtered through a 0.22 µm filter. The clear virus liquid was concentrated by 6% PEG (Sigma-Aldrich, St. Louis, MO, USA) solution in PBS (PEG MW 6000, PBS of 0.01 M at pH 7.2), and kept on ice overnight (O/N). Then the virus was pelleted by centrifugation at 2500× *g* for 30 min at 4 °C and dissolved in 2 to 5 mL of cold PBS. The final virus titer was adjusted to 4.0 × 10^8^ ffu/mL for downstream applications, such as inactivation, lyophilization and hydrogel formulation.

### 2.2. ERA-2GnRH Virus Inactivation, Lyophilization and Hydrogel Formulation

We used β-propiolactone (BPL, Sigma-Aldrich, St. Louis, MO, USA) for RABV inactivation. In brief, BPL was diluted in a chemical hood using cold, sterile water to 10% (*v*/*v*), and added to RABV ERA-2GnRH (4.0 × 10^8^ ffu/mL) to a final concentration of 0.04% (*v*/*v*). After incubation on ice for 18 h, the virus solution was further incubated in a water bath at 37 °C for 2 h. The BPL-killed virus was used immediately for lyophilization, or stored at −80 °C. For lyophilization of inactivated ERA-2GnRH virus, a benchtop freeze dryer from Labconco was used (Labconco, Fort Scott, KS, USA). One part stabilizer BSA at 10% (Sigma-Aldrich, St. Louis, MO, USA) was added to 19 parts of ERA-2GnRH, and the formula was divided into 2 mL aliquots per 5 mL vial, and was freeze-dried O/N according to the manufacturer’s program. The dry vaccine was capped under vacuum and stored at −80 °C.

For vaccine formulation in hydrogel, first a 1.2% chitosan solution in 0.1 M HCl was prepared using low molecular weight chitosan (Sigma-Aldrich, St. Louis, MO, USA). Two vials of dry vaccine ERA-2GnRH (~ 8.0 × 10^8^ ffu before inactivation) were loaded gradually into 8 mL of chitosan solution under magnetic stirring, making a vaccine at 1.0 × 10^8^ ffu/mL of hydrogel. The gel vaccine was continuously stirred at 4 °C until it was administered to mice.

### 2.3. Vaccine Gelling Studies In Vitro

Five snap cap tubes with 1 mL per tube were filled with the vaccine hydrogel prepared in Section 2.2, and 0.01% Ivan’s blue dye was added for easy observation. Tubes were incubated in a water bath at 37 °C. Every 5 min, we checked the vaccine gelling status by inverting the tube to see if the hydrogel was solidified and attached firmly to the tube wall, and the gelling time was recorded.

### 2.4. Mouse Vaccination Using the ERA-2GnRH Virus

The animal protocol 2787WUMOUC was established and followed in compliance with the CDC Institutional Animal Care and Use Committee policies. Fifty CBA/CaJ mice (Charles River Laboratory, Wilmington, MA, USA) 2-months-old, half males and half females, were divided into three groups and immunized intraperitoneal (i.p.). The i.p. route was chosen due to the large volume of vaccine delivered and the demonstrated efficacy of this route in the NIH potency test for rabies vaccines. Group A had 10 mice and served as the no-vaccination control: five males in one cage and five females in a separate cage, were injected i.p. with 0.4 mL of PBS (0.01 M, pH 7.2). Group B was the inactivated liquid vaccine group and had 20 mice: males and females housed separately with 5 animals per cage, and each mouse received 0.4 mL i.p. of inactivated ERA-2GnRH (equal to 4.0 × 10^7^ ffu before BPL inactivation). Group C was the hydrogel vaccine group and had 20 mice: half males and half females housed separately with five mice per cage, and each mouse was vaccinated i.p. with 0.4 mL ERA-2GnRH vaccine formulated in hydrogel (equal to 4.0 × 10^7^ ffu before BPL inactivation). The mice were checked twice every day, for any possible adverse reactions after injection. Blood was collected using a 4 mm Goldenrod Animal Lancet (MEDIpoint, Inc., Mineola, NY, USA) by the submandibular method on day 13, and weekly thereafter until day 47. To minimize stress on the animals, blood was collected from half of the animals in rotation every week. On day 47, mice were paired, with one female and one male in a cage. The paired animals were observed twice a day for vaginal plugs suggestive of mating. If pregnancy occurred any time after pairing, then the experiment was terminated before delivery and both of the paired animals were euthanized. Blood and gonads were collected at euthanasia for further studies.

### 2.5. Rabies Virus Neutralizing Antibodies and GnRH Antibodies in Mice

A micro-neutralizing test was applied for analyzing the RABV-neutralizing antibodies (rVNA) [13]. In each well of a Teflon-coated 4-well plate (Thermo Fisher Scientific, Waltham, MA, USA), we diluted serially 3 µL of mouse serum in 12 µL of DMEM with 10% fetal bovine serum, followed by the addition of 12 µL of RABV strain CVS-11 at 50 FFD_50_/mL. After incubation for 60 min at 37 °C in a cell culture incubator, 24 µL BSR cells at 5 × 10^5^ cells/mL were added for a further incubation of 20 h. The cells were then fixed using cold 80% acetone at −20 °C for 30 min and stained with the FITC-conjugated anti-rabies monoclonal antibodies (Fujirebio Diagnostics, Malvern, PA, USA). The serum endpoint titer was calculated using the Reed-Muench method and converted to international units (IU/mL) based on comparison to standard rabies immune globulin (US standard R-3).

The GnRH antibodies in mouse serum after immunization with ERA-2GnRH were detected using the ProteinSimple Wes with the 2–40 kDa separation module (Proteinsimple, San Jose, CA, USA). The peptide 2GnRH was synthesized at CDC, Division of Scientific Resources, and dissolved in sterile water at a final concentration of 0.2 mg/mL for Western blotting. Mouse serum was diluted 1:5 in the PBS (0.01 M, pH 7.2) before loading in the ProteinSimple Wes.

### 2.6. Statistics

For rabies virus-neutralizing antibody, the geometric mean and standard deviation were calculated for each group of animals. GraphPad Prism (v 6.07) was used for non-linear curve fit of rabies virus neutralizing antibody data using a one-phase association exponential equation and to calculate p-values using a two-tailed Fisher Exact test.

## 3. Results

### 3.1. The ERA-2gnrh Vaccine Was Formulated in a Thermo-Responsive Chitosan Hydrogel

**Figure 1:**
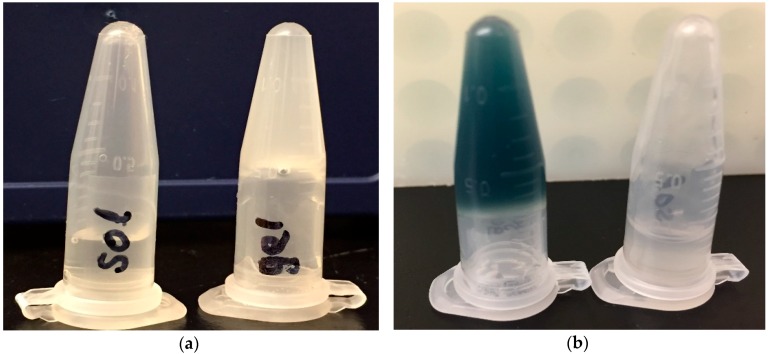
Gelling study of the ERA-2GnRH hydrogel vaccine. (**a**) Tube 1 is the ERA-2GnRH hydrogel at room temperature; the gel is liquid and settles down to the bottom. Tube 2 is the hydrogel vaccine incubated at 37 °C for 10 min. (**b**) Tube 1 is the hydrogel vaccine with a blue dye (0.01% Ivan’s blue) incubated at 37 °C for 10 min. Tube 2 is the room temperature product, a liquid gel.

To create an “antigen depot effect” for an enduring immune stimulation, we loaded dry ERA-2GnRH vaccine into a chitosan solution. Viscosity is one concern for chitosan hydrogel needle injection; where, a high gel concentration retains antigen longer but is difficult to inject. To optimize the vaccine formulation by balancing antigen retention and gel viscosity, we concentrated and freeze-dried the virus, and loaded it into a 1.2% chitosan hydrogel, a concentration we pretested for mouse i.p. injections (data not shown). The formulated ERA-2GnRH chitosan hydrogel vaccine was liquid O/N at 4 °C, remained liquid at R/T (22 °C ± 3 °C) for ~1 h, and became a semi-solid gel at 37 °C between 5 and 10 min (Table 1). The gelling was visualized by adding a blue dye and simply inverting the tube, with the gel sticking to the top of the inverted tube (Figure 1). Using a 3 mL syringe with a 23-gauge needle, the formulated ERA-2GnRH vaccine was drawn and pushed out freely. The final vaccine for mouse inoculation was a 1.2% chitosan solution, loaded with 1.0 × 10^8^ ffu/mL ERA-2GnRH in the thermo-responsive hydrogel.

### 3.2. The Chitosan Hydrogel ERA-2GnRH Vaccine Induced High and Persistent Rabies-Neutralizing Antibodies in Mice

The adjuvant properties of chitosan have been shown in many previous reports [14,15,16]. We chose i.p. rather than s.c. injection because of the requirements of the NIH test for potency of inactivated rabies vaccines [17,18,19]. Each mouse in Groups B and C received 0.4 mL of vaccine approximately 4.0 × 10^7^ ffu of virus before inactivation. A subcutaneous mass on the lower abdominal area of mice 2–3 days after administration was observed, suggesting vaccine solidified in the animal body. The mass dissolved progressively, and the mice did not show signs of pain or distress, and gained normal weight in the course of the experiment (data not shown).

**Figure 2:**
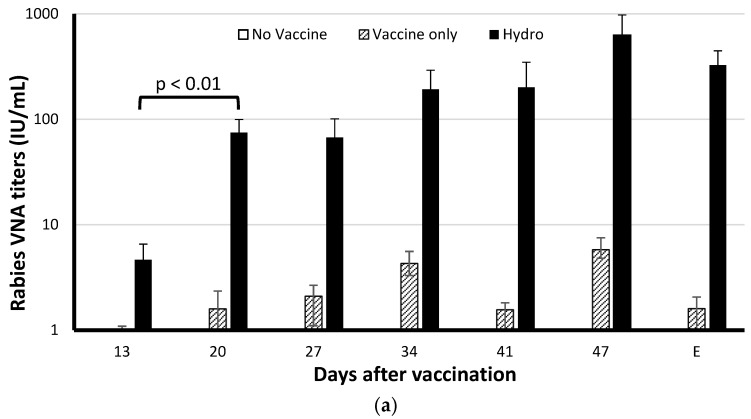

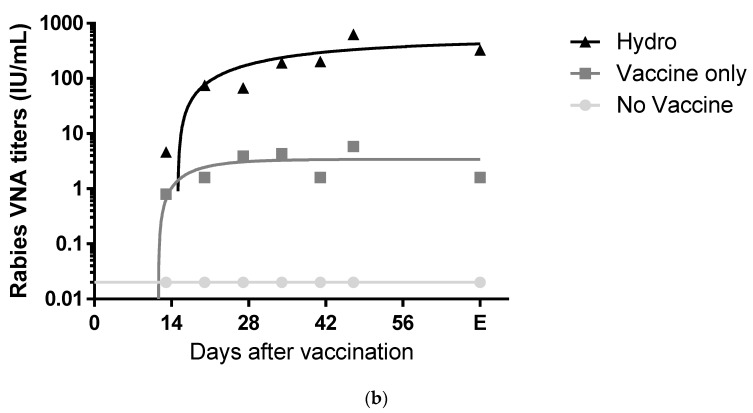
Dynamics of rabies virus-neutralizing antibody titers after immunization in mice. (**a**) Geometric mean titer with standard deviation and (**b**) non-linear curve fit for each group at each time point are shown on a log-scale. Mice in Group A (*n* = 10) received no vaccine. Mice in Group B (*n* = 20) received inactivated ERA-2GnRH (Vaccine only). Mice in Group C (*n* = 20) received an equivalent dose of inactivated ERA-2GnRH in a chitosan hydrogel (Hydro). The end of the study (E) was approximately 70 days post-vaccination but varied for each group based on fertility.

Using a cutoff of 0.1 IU/mL for seroconversion [13], all mice were rVNA negative in Group A at all time points, <0.02 IU/mL (Figure 2). On day 13, all mice in Groups B and C seroconverted. For Group B, the rVNA ranged from 0.17 IU/mL to 2.62 IU/mL, and the geometric mean titer (GMT) was 0.80 IU/mL. In Group C, the rVNA ranged from 0.52 IU/mL to 20.73 IU/mL, and the GMT was 4.60 IU/mL. Chitosan in the hydrogel vaccine did not induce an early rabies immune response, but the adjuvant activity of chitosan enhanced the GMT by more than 5-fold compared to the vaccine only group (Figure 2a). On day 20, the rVNA for Group B varied from 0.15 IU/mL to 8.16 IU/mL, with the GMT of 1.60 IU/mL. In Group C, the rVNA increased significantly compared to day 13 (*p* < 0.01), with a GMT of 74.80 IU/mL. The results demonstrated a strong anti-rabies response that occurred within 3 weeks, similar to previous studies with inactivated rabies vaccines [20,21]. As shown in Figure 2a, the rVNA in Group A mice was always negative, <0.02 IU/mL; while the mean rVNA in Group B mice increased until day 47, and then subsided, but was still above the 0.5 IU/mL threshold, an arbitrary level representing adequate rabies vaccination. In Group C, the mean rVNA persisted above 300 IU/mL up to the termination of the animal study on day 70 (Figure 2). Based on the non-linear curve fit (Figure 2b), vaccine ERA-2GnRH in the liquid formulation had a fast release profile with a half-time of approximately 5 days; while the ERA-2GnRH in the hydrogel formulation had a slow, controlled release process with a half-time of approximately 20 days. Both administrations induced an adequate rabies immune response 13 days after immunization.

### 3.3. The Liquid Vaccine ERA-2GnRH Induced A Fast GnRH Antibody Response in Mice

**Figure 3:**
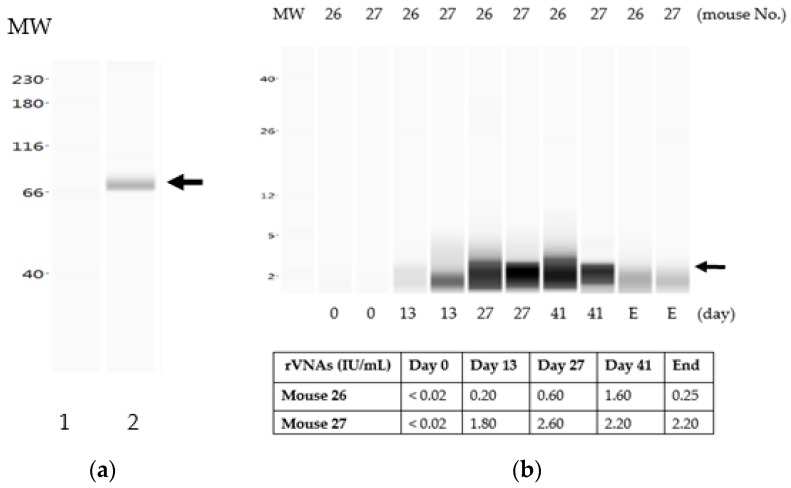
Antibody dynamics of GnRH in mice vaccinated with the liquid ERA-2GnRH vaccine. Protein detection using the ProteinSimple Wes. (**a**) Detection of recombinant protein G-2GnRH using antibodies against GonaCon; lane 1, molecular weight (MW) marker; lane 2, recombinant G-2GnRH protein from purified ERA-2GnRH virus; (**b**) GnRH antibodies in representative mice receiving the liquid ERA-2GnRH vaccine over time; GnRH antibodies were detected in mouse 26 and 27 (Group B) from day 13 through day 41 and then decreased until the end of the experiment (E, ~day 70). Similarly, the rVNA titer was positive on day 13, went up on days 27 and 41, and declined until E.

We chose two mice from the vaccine only group to project the trend of GnRH antibodies after vaccination from day 0 through the end of the experiment. Before the testing, we first developed a Western blotting method for detecting GnRH antibodies in mice. To evaluate the method, purified RABV ERA-2GnRH was loaded in the ProteinSimple Wes, a transfer-free automated Western blotting system, and anti-GnRH antibodies (rabbit against GonaCon) were used for detection. In Figure 3a, a single clear band was detected, corresponding to the size of recombinant glycoprotein G-2GnRH (~66 kDa). Therefore, the ProteinSimple Wes was chosen to detect GnRH antibodies in the vaccinated mice. In Figure 3b, the GnRH antibody was barely detectable on day 13, corresponding to the time when rVNAs seroconverted (Figure 2). The GnRH band in the ProteinSimple Wes going from dark (on day 27 and day 41) to faint (on E, end of the experiment ~day 70) also correlated to the dynamics of rVNAs. We concluded there was a concurrent immune response between GnRH antigen and its vector RABV in the liquid vaccine formulation. The vaccine released fast after injection and induced a fast immune response, but declined after day 41.

### 3.4. The Chitosan Hydrogel ERA-2GnRH Vaccine Induced a Slow, but Sustained GnRH Immune Response in Mice

**Figure 4:**
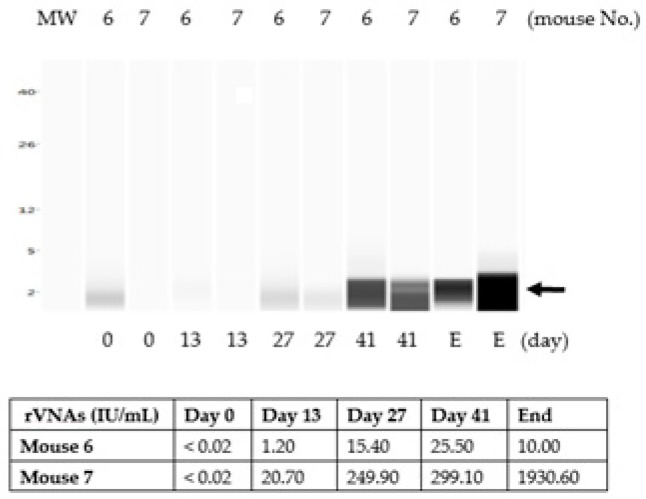
GnRH antibodies in mice immunized with ERA-2GnRH hydrogel. The GnRH antibodies were delayed and detected from day 41 through the termination of the experiment around day 70, which is suggestive of a slow, controlled vaccine release profile.

In contrast to the liquid vaccine formulation in group B, the ERA-2GnRH in hydrogel induced a slow immune response against GnRH. The GnRH band by ProteinSimple Wes was clearly visible only on day 41, 4 weeks later than the liquid vaccine (Figure 4). Interestingly, when the GnRH antibodies subsided in Group B (Figure 3b), the GnRH antibodies increased in Group C, suggesting a slow-release profile in the hydrogel vaccine. The GnRH antibodies were sustained until the end of the experiment at approximately day 70 (Figure 4).

### 3.5. The GnRH Antibodies in Mice Were Not Sufficient to Prevent Pregnancy by One-Dose Vaccination

**Figure 5:**
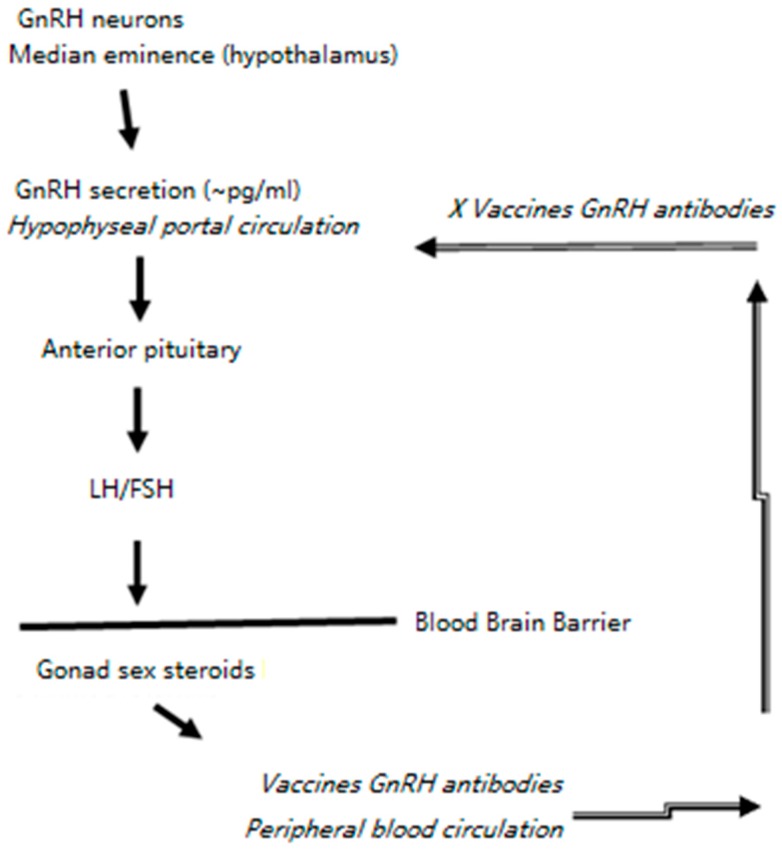
Mechanism of GnRH-based antibodies on contraception. GnRH neurons in hypothalamus secrete GnRH to the hypophyseal portal system (HPS). Free GnRH in the HPS stimulates the release of luteinizing hormone (LH) and follicle-stimulating hormone (FSH) in the anterior pituitary. After crossing the blood brain barrier, the LH and FSH work on the gonads and stimulate the reproduction cycle. The GnRH antibodies after vaccination with ERA-2GnRH bypass the Blood Brain Barrier through the HPS and catch/neutralize free GnRH in the median eminence, thus blocking the downstream signaling for reproduction.

On day 47 post-vaccination, mice were co-housed as one female mouse with one male mouse within each group for breeding. The CBA/CaJ mouse model was efficient and convenient in our previous study in evaluating the immunocontraceptive vaccine [11]. Within 10 days after being paired, all female mice in the unvaccinated Group A became pregnant. In Groups B and C, within a month, all the female mice also became pregnant (data not shown). That mice in Group B were fertile was not unexpected, since the GnRH antibodies were waning 41 days after vaccination (Figure 3b). However, we were surprised the GnRH antibodies in Group C mice were also not high enough to prevent pregnancy. We think the GnRH antibodies could have a threshold level in the peripheral blood in order to reach the hypophyseal portal system in the brain for fertility disruption (Figure 5). Also, the live ERA-2GnRH vaccine we tested previously could be different from the inactivated vaccine in inducing infertility [11].

## 4. Discussion

Our study for the first time demonstrated that inactivated rabies vaccine formulated in hydrogel could induce a high and long-lasting immunity against rabies. This fortuitous result will direct our future studies in exploring the possibility of one-dose vaccination in animals, or even humans. Rabies prophylaxis in humans is not simple, including pre-exposure prophylaxis (PrEP), post-exposure prophylaxis (PEP), and re-exposure prophylaxis (REP). Vaccination follows strict schedules recommended by WHO and ACIP according to the vaccine type and administration route. The awareness of dog rabies as a major threat to humans has changed little over thousands of years since early human civilization [22,23]. The simple and strong message in rabies control is to “vaccinate dogs”. The primary reason rabies still kills ~60,000 people each year worldwide is insufficient dog vaccination due to a lack of commitment, limited resources, and technical challenges associated with vaccination. Currently, the burden of rabies deaths is mainly in Asian and African countries where limited resources are available for rabies control, and the recommended dog vaccination schedule is not easy to follow, if not impossible. Our intention is to develop a vaccine that protects dogs from rabies for more than 3 years in a single dose, an ambition which has not previously been achieved, or seriously attempted. One goal of the current study on ERA-2GnRH was to test if the vaccine formulated in hydrogel could induce a long-lasting immune response. Our rationale is that rabies vaccine in the hydrogel releases slowly after delivery, and the adjuvanticity of chitosan will further boost the immune responses [14,15,16]. In the pioneering study using the human diploid cell rabies vaccine in 1976 [24], vaccine without any adjuvant was administrated subcutaneously to the patients after rabid dog or wolf bites in Iran on days 0, 3, 7, 14, 30, and 90. After 32 years of the initial PEP, 26 patients in the follow-up investigations had detectable rabies antibodies, and one vaccine booster induced strong anamnestic responses in all the subjects [25]. Another study extrapolated from the longevity of rabies titers and suggested subjects with rVNA ≥30 IU/mL could be protected from rabies for at least 10 years [26]. The longevity of rabies immunization has been repeatedly reported [27,28,29]. However, there is no study or suggestion that PEP or PrEP generates full rabies protection if the patient is re-exposed to rabies without any further prophylaxis, i.e., REP on days 0 and 3 is still required. The take-away message from the longevity studies in humans is that rVNA persists and the immune memory is long.

The chitosan hydrogel may help induce a continuous rVNA response over time. We observed in mice that the ERA-2GnRH hydrogel vaccine was absorbed and no lesions were observed at the injection site during necropsy when the experiment ended. Chitosan has a safe profile in administration, has been used as a food supplement, and is being used in the pharmaceutical industry for drug delivery and tissue engineering [30,31,32]. The adjuvanticity of chitosan has also been demonstrated in many studies [14,15,16] with the transition from a liquid state to a semisolid hydrogel creating an “antigen depot” effect after injection into the animal, and thus the ERA-2GnRH induces a long-lasting immunity. In our study, the vaccine in hydrogel generated persistent and high rVNA from day 13 until the experimental endpoint. The 20 mice in the hydrogel group (Group C) had rVNA of 327.40 IU/mL compared to 1.60 IU/mL in the vaccine only group (Group B) at the experimental endpoint. This data supports the concept that ERA-2GnRH vaccine in chitosan hydrogel could induce longer rabies protection in dogs than traditional rabies vaccine.

To combine a “fertility-control vaccine” with a rabies vaccine without changing the rabies vaccination schedule will be an added advantage for dog rabies control. On a regular schedule, a dog receives two rabies vaccinations in the first year (on day 0 and day 365) and a booster every year or every 3 years thereafter. If our vaccine ERA-2GnRH follows this regimen by receiving two vaccinations in the first year and a booster every year thereafter, the dog could be infertile after such multiple vaccinations, with no need for additional surgical spay/neuter in the veterinary practice. The previous study in mice demonstrated that after three vaccinations using the ERA-2GnRH, the animals were permanently infertile [11]. It would be worthwhile to confirm whether the same might be true in dogs.

There is a broad interest in finding a non-surgical fertility control method for free-ranging animals, shelter dogs and cats, pest animals, overpopulated animals, and zoo animals [33,34,35,36,37,38]. Under experimental conditions, many investigations achieved temporary and reversible infertility in animals using GnRH analogs, or antagonists, including antibodies against GnRH [39,40,41,42,43,44,45]. The mechanism of how GnRH antibodies impair animal reproduction is depicted in Figure 5. The idea of using an immune contraceptive vaccine for animal population control started about half a century ago with porcine zona pellucida (PZP), a raw glycoprotein mixture exacted from pig ovaries [46,47,48,49]. PZP vaccination prevents sperm from binding to oocytes, and thus is only appropriate for contraception in female animals. The United States Environmental Protection Agency licensed the PZP vaccine ZonaStat-H and GnRH vaccine GonaCon for use in wild horses and burros [50,51]. With the help of strong adjuvants (Freund’s incomplete adjuvant FIA in ZonaStat-H, and Adjuvac in GonaCon) and high protein/antigen concentrations, multi-year infertility in animals was achieved after GonaCon immunization [52,53]. However, we know antibodies after immunization will peak during a time window, subside, and eventually become undetectable in months or years. “Developing a permanent, single-dose non-surgical sterilant for male and female cats and dogs” is a challenge goal that the Alliance for Contraception in Cats & Dogs has been leading since 2000 [54].

Using the ERA-2GnRH vaccine, our goal is to combine rabies control and dog population management. Culling dogs for rabies control is not only controversial but also ineffective [55,56]. A dual vaccine that protects against rabies and controls the dog population would be a significant advancement for rabies control programs. We should not ignore the fact that there is no licensed contraceptive vaccine available for dogs, and the “catch-neuter-vaccinate and release method” can reach only a very limited number of animals, thus having a limited impact on animal population control [57,58]. Our technical interest is to improve ERA-2GnRH by formulating the vaccine in a chitosan hydrogel. The slow-releasing formulation induced persistent GnRH antibodies that lasted up to the end of the experiment at day 70 (Figure 4). Without hydrogel, the GnRH antibodies appeared on Day 13 and faded after Day 41 (Figure 3b). Therefore, the ERA-2GnRH vaccine in hydrogel achieved our goals in the study and induced high and sustainable antibodies against both rabies and GnRH. However, the observation that the animals were fertile after vaccination adds an extra layer of complexity as to how GnRH antibodies prevented fertility in our previous studies [11]. This suggests a threshold level of anti-GnRH antibodies, as proposed previously [59], was not achieved in the current study, or a replication competent vector may be required to induce infertility. For future studies, we will design a strategy to ensure sufficient and sustained anti-GnRH antibodies after immunization. With additional improvements in vaccine formulation and delivery, we believe the ERA-2GnRH vaccine can be an efficacious dual vaccine for rabies protection and dog population management.

## Figures and Tables

**Table 1:** Thermo-responsive hydrogel vaccine ERA-2GnRH.

Measurement	ERA-2GnRH	Chitosan
Titer/Concentration	1.0 × 10^8^ ffu/mL	1.2%
Room Temperature	Stable in lyophilized form	60 min
37 °C	Not tested	5–10 min
4 °C	Stable	Overnight
